# MIS-C: Diagnosis, Management, and Outcomes

**DOI:** 10.1093/ofid/ofaf762

**Published:** 2025-12-19

**Authors:** Christophe El Rassi, Roy El Darzi, Maria Abou Mansour, Mariam Arabi

**Affiliations:** Faculty of Medicine, American University of Beirut Medical Center, Beirut, Lebanon; Faculty of Medicine, American University of Beirut Medical Center, Beirut, Lebanon; Faculty of Medicine, American University of Beirut Medical Center, Beirut, Lebanon; Faculty of Medicine, American University of Beirut Medical Center, Beirut, Lebanon; Pediatric Department, Division of Pediatric Cardiology, American University of Beirut Medical Center, Beirut, Lebanon

**Keywords:** COVID-19, immunomodulatory, MIS-C, therapy

## Abstract

Multisystem inflammatory syndrome in children (MIS-C) is an emergent postinfectious hyperinflammatory disorder predominantly affecting the pediatric population following COVID-19 infection. Clinically, it is characterized by persistent fever, shock, multiorgan involvement, and potentially severe cardiovascular involvement. This comprehensive review synthesizes current evidence on the epidemiology, pathophysiology, clinical presentation, diagnostic criteria, with particular emphasis on the management of MIS-C. We also stress on the importance of distinguishing MIS-C from phenotypically similar entities. Acute-phase management centers on supportive care, hemodynamic stabilization, and targeted immunomodulation, with intravenous immunoglobulin, corticosteroids, and biologic forming the therapeutic cornerstone. Thromboprophylaxis is frequently warranted due to the elevated thromboembolic risk, and long-term follow-up is essential to monitor for cardiac, gastrointestinal, and neurologic complications. Additional considerations include postrecovery vaccination protocols and the use of extracorporeal membrane oxygenation in cases of refractory cardiorespiratory failure. Despite advancements in clinical outcomes, diagnostic ambiguity and heterogeneous management guidelines continue to pose significant challenges.

## Teaser

This review outlines current strategies for diagnosing and managing multisystem inflammatory syndrome in children (MIS-C), emphasizing clinical features, differential diagnosis, immunomodulatory treatment, and long-term outcomes, while highlighting knowledge gaps and variations across global health guidelines.

In 2020, amidst the COVID-19 pandemic, multisystem inflammatory syndrome in children (MIS-C) emerged as a post-SARS-CoV-2 (severe acute respiratory syndrome coronavirus 2) complication in the pediatric population [1, 2]. Initially termed PIMS-TS (pediatric MIS, temporally associated with COVID-19), it was then labeled by the Centers for Disease Control and Prevention (CDC) as MIS-C [3, 4]. Multiple definitions for MIS-C were proposed by different organizations such as the CDC [5] and Royal College of Pediatrics and Child Health (RCPCH) [6], which all included fever, involvement of at least one organ system, and inflammation as typical diagnostic findings in a child previously infected with SARS-CoV-2. While the mortality of COVID-19 was reported to not exceed 0.2 per 100,000 population that of MIS-C ranges between 1 and 2 per 100 cases [7–9]. This highlights the importance of proper management of MIS-C. This disease also presented itself as having many clinical similarities to other inflammatory conditions such as Kawasaki disease (KD), culture negative sepsis, and microbial infections like rickettsiosis or typhoid fever [10–13]. The first cases of MIS-C reported in the literature consisting of 8 children and adolescents presenting with a hyperinflammatory syndrome in April 2020, were described as showing characteristics of atypical KD [14].

The exact pathogenesis of MIS-C remains a mystery, although the immune system is known to be a major protagonist in this postinfectious disease [15]. Until now, a plethora of management modalities have been used and proposed, ranging from glucocorticoids to immunomodulatory agents in addition to anticoagulation therapy [9]. Other exceptional approaches such as extracorporeal membrane oxygenation (ECMO) might be employed in more severe cases of MIS-C [16, 17]. Although many protocols and treatment guidelines were proposed by various societies such as the American Academy of Pediatrics [18], American College of Rheumatology (ACR) [19], and Canadian Pediatrics Society [20], there is still a lack of consensus on a single treatment system [17]. This review provides an overview of MIS-C, discussing its epidemiology, pathogenesis, clinical presentation, and differential diagnoses, with a focus on the use and efficacy of the management modalities employed.

MIS-C is now defined as a delayed, hyperinflammatory condition occurring typically 2 to 6 weeks after acute SARS-CoV-2 infection or exposure, characterized by fever, laboratory evidence of inflammation, and multisystem (≥2 organs) involvement without an alternative plausible diagnosis [5, 21] (CDC, 2023; WHO, 2020). A fever >38.0°C lasting more than 24 hours is required for diagnosis, accompanied by elevated inflammatory markers such as C-reactive protein, ferritin, D-dimer, and erythrocyte sedimentation rate [5, 21]. The unifying feature among these diverse presentations is a postinfectious immune dysregulation that leads to systemic inflammation, often involving cardiac, gastrointestinal, neurologic, and hematologic systems [22–24]. MIS-C typically lasts 1 to 2 weeks with appropriate treatment, though cardiac and gastrointestinal manifestations may persist longer [25, 26]. Reported mortality is ∼1%–2%, and the majority of patients recover fully with timely immunomodulatory therapy [24, 27].

## METHODS

A comprehensive literature search was performed between February and May 2025. The review was conducted on PubMed and Google Scholar using the keywords: MIS-C—“Multisystem” “inflammatory syndrome in children”—“PIMS-TS” combined with “diagnosis”—“pathophysiology”—“treatment”—“management”—“steroids”—“biologics”—“IVIG” (intravenous immunoglobulin)—“hospitalization” for our search. Articles addressing the diagnosis, pathophysiology and management modalities of MIS-C were gathered, and their findings were analyzed then summarized in our review. Accuracy was confirmed through cross-referencing findings with other sources, while validity was determined by critical appraisal tools relevant to their respective designs: observational studies were evaluated using the Newcastle–Ottawa Scale, and qualitative studies were assessed using the Critical Appraisal Skills Programme checklist. Relevance was then assessed by matching the studies' findings with our review objectives. No limitations on the type of studies or country of origin were made, but articles were referenced only if written or translated in English. The search was done within the 2020–2025 period. Other papers were included from the references of the articles chosen.

## RESULTS

### Epidemiology

MIS-C is a rare but serious inflammatory syndrome that develops 2 to 6 weeks following SARS-CoV-2 infection [28]. Studies conducted in the United States during the early phase of the COVID-19 pandemic (April to June 2020) showed an incidence of approximately 5 MIS-C cases per million person-months or 316 cases per million SARS-CoV-2 infections among individuals under 21 years of age [29]. Moreover, a study in England including children aged 0 to 16 years, found that the Delta and Omicron variants of SARS-CoV-2 were associated with a lower risk of MIS-C compared with the Alpha variant [30]. Cohen et al found that the reduction in MIS-C incidence observed during the Delta variant is unlikely to be solely attributed to immunization, either prior infection or vaccination, but rather to alterations in viral epitopes responsible for triggering hyperinflammation [30]. As for the Omicron variant, specific spike protein substitutions such as N679K may attenuate its superantigen-like properties, consequently decreasing its propensity to trigger MIS-C [30].

MIS-C has been reported across a wide age range, from 1 month to 21 years but is most frequently observed in children aged 6 to 12 years [31–34]. According to several systematic reviews, the median age of affected children typically falls between 8 and 9 years [31, 35, 36]. A MIS-C-like syndrome has also been observed in a limited number of young adults, referred to as MIS-A [37]. Most studies report a male predominance, with a meta-analysis by Guimarães et al showing that 62% of MIS-C cases occurred in males, resulting in a male-to-female ratio of 1.6:1 [31, 35, 38]. The majority of children with MIS-C were previously healthy, with over half of the patients having no pre-existing conditions [31, 36]. The temporal and clinical evolution of MIS-C across the COVID-19 pandemic is summarized in Table 1, which outlines representative studies from 2020 to 2025 detailing patient demographics, symptom patterns, comorbidities, and outcomes.

**Table 1.** Overview of Major MIS-C Studies (2020–2025), Including Age Distribution, Symptom Frequency, Comorbidity Prevalence, Hospitalization Outcomes, and Mortality, Illustrating Changes in Disease Phenotype and Prognosis Throughout the Pandemic

Year	Study Type	Sample Size	Age (mean/median)	Common Symptoms (Fever, GI, Cardiac, and Mucocutaneous)	Pre-existing Conditions	Hospital Stay (median)	Mortality
2020 [24]	Multicenter observational cohort (CDC)	186 children	8.3 y (median)	Fever in ∼100% (criterion); GI 92%; cardiac 80%; mucocutaneous 74%	73% previously healthy.	7 d	2% (4 deaths)
2020 [33]	Statewide surveillance descriptive study	95 children (confirmed MIS-C)	∼11 y (median; IQR 8–14)	Fever 100%; GI symptoms 80%; cardiac (myocarditis/shock) ∼53%–62%; rash 60%, conjunctivitis 56%	67% previously healthy.	6 d	∼2% (2 deaths)
2021 [34]	Multicenter comparative cohort (MIS-C vs severe COVID)	539 MIS-C (vs 577 COVID)	8.9 y (median MIS-C)	Fever 99%; GI 90%; mucocutaneous 67%; respiratory 43%	∼69% with no underlying conditions.	7 d	1.9% (10 deaths)
2021 [39]	Multicenter prospective cohort (17 PICUs in Brazil)	56 children	6.2 y (median; IQR 2.4–10.3)	Fever 100% (inclusion); GI symptoms 71%; shock 59% (hypotension); severe respiratory <20%. Many had Kawasaki-like illness (47% overall).	80% had no comorbidities.	6 d in PICU	1.8% (1 death)
2022 [40]	Prospective population-based cohort	52 MIS-C cases (Aug 2021–Feb 2022)	∼N/A (ages 0–17; most school-age)	Clinical features during the Delta wave mirrored early pandemic MIS-C, with universal fever and predominant gastrointestinal and cardiac involvement.	∼98% unvaccinated; high proportion previously healthy.	N/A	0% (no deaths reported in cohort)
2022 [41]	Nationwide cohort (risk-factor analysis)	253 MIS-C cases (Mar 2020–Dec 2021)	Peak incidence in 5–11 y-olds (incidence 6.8 per 100k person-years)	Majority had fever and multiorgan inflammation; many cases noted Kawasaki-like signs.	>80% had no significant comorbidities.	N/A	<1%
2023 [42]	Multinational EHR-based cohort (variant-era comparison)	Presumably ∼600–800 MIS-C cases across 4 countries (USA and Europe)	∼N/A (overall median not given; Omicron-era cases skewed younger)	Delta versus Omicron: Marked shift in symptoms—Omicron-era MIS-C cases had fewer shock/cardiac complications and milder fever/Kawasaki-like illness compared with earlier variants	No major change in comorbidity profile over time	Shorter ICU stays in Omicron-era (not explicitly quantified)	Overall mortality remained low not explicitly stated; implied <2%)
2023 [43]	National surveillance comparison (Alpha/Delta vs Omicron)	91 children (2020–Apr 2022)	Median ∼10 y pre-Delta; ∼9 y Delta; ∼7 y Omicron (trend toward younger age in Omicron wave)	Pre-Delta versus Omicron: Fever ∼100% → ∼100%; GI involvement 100% → 6%; Shock/hypotension 67% → 12%; ICU admission 50% → 10.6%. (Omicron MIS-C had far fewer shock and GI symptoms than earlier cases)	Most had no comorbidities	ICU stay dropped dramatically by Omicron	0% (no MIS-C deaths reported in SK up to 2022)
2024 [44]	Multicenter cross-sectional (2021 vs 2023 comparison)	3578 (2021) versus 221 (2023) MIS-C hospitalizations	2021: 9 y (median); 2023: 6 y (median)	2021: ∼100% fever, predominant GI and shock presentations (baseline). 2023: illness shifted to younger ages with more Kawasaki-like mucocutaneous features	>50% had no comorbidities in both years	2021: median 7 d; 2023: median 5 d; ICU admission rate ∼45% in 2023 versus ∼>50% in 2021	2021: 0.6% (22 deaths) versus 2023: 2.3% (5 deaths)
2025 [45]	Systematic review & network meta-analysis	69 studies, >6500 MIS-C cases	Pooled median ∼8 y	Fever ∼100%; GI > 80%; cardiac ∼70%; mucocutaneous ∼60%	>70% previously healthy	6–8 d	1.6% overall

### Pathophysiology and Clinical Presentation

#### Pathophysiology

As indicated by its early nomenclature “PIMS-TS,” MIS-C is a complication observed in children following COVID-19 [4]. To date, the exact pathogenesis MIS-C remains unknown, and different theories are postulated. Post-COVID-19 immune dysregulation leading to a “cytokine storm” is one such theory. A molecular profiling study by Diorio et al analyzed plasma proteoms from children with MIS-C, severe COVID-19, and post-COVID-19 controls [24]. They identified that CXCL9, a chemokine induced by IFN-γ, showed a disproportionately greater expression in response to IFN-γ in MIS-C patients compared with non-MIS-C cohorts, suggesting a cytokine amplification cascade at the center of MIS-C pathogenesis [46]. Of note, it is thought that the pathogenesis of MIS-C is not dependent on direct viral cytotoxicity, but is rather driven by an indirect immune-mediated response [47], such as dysregulation or overactivation following COVID-19 [48].

 A cytokine storm describes a dysregulated hyperinflammatory reaction in which immune signaling escapes normal feedback control, resulting in excessive cytokine release and systemic injury [49]. In the context of SARS-CoV-2-related inflammation, rapid activation of Th1 cells and monocytes drives increased production of IL-6, tumor necrosis factor α (TNF-α), interferon gamma (IFN-γ), as well as IL-1β, IL-8, IL-10, IL-15, IL-17, and IL-18 [15, 46, 50].They identified that CXCL9, a chemokine induced by IFN-γ, showed a disproportionately greater expression in response to IFN-γ in MIS-C patients compared with non-MIS-C cohorts, suggesting a cytokine amplification cascade at the center of MIS-C pathogenesis [46].

Viral engagement of pattern recognition receptors, disruption of angiotensin pathways, and suppressed early interferon responses amplify this process by preventing timely transition from innate to adaptive immunity [50, 51]. In turn, this results in a reinforced inflammatory loop where proinflammatory mediators and cytokines are overproduced, which gives rise to the cytokine storm image observed in MIS-C. One third of the patients presented with this pattern of intense cytokine release [52]. This hyperinflammatory trend is consistent in MIS-C, as an early observational study during the COVID-19 pandemic found that more than 90% of the patients presented with elevations of 4 or more inflammatory biomarkers [24]. While no specific cytokine threshold has been defined that predicts the onset of MIS-C within a certain post-COVID-19 population, several studies support an association between heightened inflammatory mediator levels and more severe prognosis. In a prospective cohort by Lawrence et al, children with MIS-C exhibited significantly higher IL-6, IL-8, IL-10, and IL-13 levels than the acute COVID-19 cohort, and these elevations paralleled increased cardiac and systemic inflammation markers such as CRP and pro-BNP [53]. Similarly, Klocperk et al demonstrated a significant elevation of IFN-γ in MIS-C patients compared with healthy post-COVID-19 children, a finding that may reflect persistent immune activation even after viral clearance in MIS-C [54].

The cytokine storm (extensive inflammation) as well as other downstream inflammatory molecules it can induce, endothelial injury due to deposition of immune complexes and resultant complement activation, are possibly what leads to the multiorgan damage aspect of the disease [15, 46, 51]. By the same token, SARS-CoV-2 (and MIS-C) are marked by a transient but pronounced hypercoagulable state driven by this endothelial injury and platelet hyperreactivity [55]. The damaged endothelium and neutrophil extracellular traps (NETs), commonly seen as part of SARS-CoV-2's pathogenesis [50, 51, 56], promote coagulation pathways via release of tissue factor, factor XII, vWF, and other prothrombotic molecules [55]. Clinically, this is reflected by elevated D-dimer and fibrinogen levels and thromboelastographic findings consistent with increased clot strength, faster clot formation, and delayed fibrinolysis [55, 57]. Therefore, beyond the hyperinflammatory response, the accompanying hypercoagulable state likely contributes to end-organ injury [51].

A recurrent theme we will explore in MIS-C pathophysiology is the presence of cardiovascular damage, being at the root of widespread organ injury. This occurs through the interaction between complement and circulating immune complexes, ultimately leading to endothelial injury [15]. Further, the activation of CX3CR1^+^ CD8^+^ T-Cells, a specific feature of MIS-C, is suggested to promote cardiovascular abnormalities [48]. A molecular profiling study by Vella et al established a possible positive relationship between the activation of these cells and the presence of vascular complications [58].

#### Clinical Presentation

The typical presentation of MIS-C is fever accompanied by a multisystemic manifestation, shock, and, in severe cases, death [1, 59, 60]. Although COVID-19 results in low mortality rates in the pediatric population, MIS-C presents a 10-fold increased fatality risk compared with COVID-19 in this age group [15, 59]. Although MIS-C usually affects children, it can occur in any age group under different variations of the disease (MIS-N in neonates [61]; MIS-A in adults [62]). A slight male predominance is reported in the literature [15, 60]. Organ involvement is widespread throughout the body, and MIS-C can affect the gastrointestinal, hematologic, neurologic, respiratory, mucocutaneous, and cardiovascular systems [34, 60, 63] (Table 2).

**Table 2.** Clinical Features of Multisystem Inflammatory Syndrome in Children (MIS-C) Across Body Systems

Body System	Multisystem Inflammatory Syndrome in Children (MIS-C)
Cardiovascular	Myocarditis Coronary artery dilatation or aneurysms Shock Decreased heart function
Gastrointestinal	Abdominal pain Vomiting Diarrhea Pseudo-appendicitis Elevated liver enzymes
Neurologic	Stroke Encephalopathy Headache Altered mental status Seizures
Pulmonary	Respiratory insufficiency Pulmonary edema Pleural effusion Cough Hypoxemia
Mucocutaneous	Rash Conjunctivitis (rarely) Red cracked lips Strawberry tongue
Hematologic	Coagulopathy Elevated D-dimer levels Thrombocytopenia Elevated inflammatory markers
Renal	Acute kidney injury Proteinuria Hematuria
Musculoskeletal	Myalgia Arthralgia Arthritis

Gastrointestinal symptoms are the most commonly reported in MIS-C after fever, with around 71% to 90% of patients presenting with either diarrhea, abdominal pain, nausea, vomiting, and anorexia [34, 60, 64]. Other symptoms commonly seen as manifestations of the disease include maculopapular rashes, oral mucocutaneous discoloration [65], headache, irritability [60], acute kidney injury [66], respiratory distress, and cough [63, 64]. Furthermore, cardiac involvement is a prominent characteristic of MIS-C, occurring in around 60–80% of the cases [60, 63, 64]. Notorious findings are CAA (coronary artery aneurysms) [64, 67], ventricular dysfunction requiring inotrope use [64], and heart block [67]. In more severe cases, severe shock might lead to death [67]. MIS-C presents clinically as a close mimic to KD and toxic shock syndrome (TSS) [68]. However, the average age of MIS-C patients is typically higher than that of KD patients but lower than that of TSS patients, regardless of the causative organism of TSS [69, 70].

### Diagnostic Criteria and Challenges to Management

The diagnostic criteria for MIS-C are nonspecific and can overlap with a range of other infectious and noninfectious conditions, necessitating a thorough evaluation of alternative diagnoses [71]. The CDC, World Health Organization (WHO), and RCPCH all emphasize the importance of excluding other potential causes before establishing a diagnosis of MIS-C, as shown in Table 3. Differential diagnosis for MIS-C includes other inflammatory conditions such as KD, TSS, and sepsis [73, 74]. KD presents as an acute, systemic vasculitis of unknown etiology, more common in Asia (while MIS-C is not), and generally affects younger children and infants, typically under 5 years [75]. It is also less often associated with gastrointestinal symptoms and cough and rhinorrhea compared with MIS-C [75]. TSS is characterized by a cytokine storm, triggered by superantigens primarily produced by *Staphylococcus aureus* and *Streptococcus pyogenes* [76]. Sepsis involves a systemic inflammatory response to infection, which can be life-threatening and may present with features similar to MIS-C [77].

**Table 3.** **Case Definitions of MIS-C** ^a,b,c^

Criteria	RCPCH (2021)	CDC (2023)	WHO (2020)
Age	Not defined	<21 y	<19 y
Fever	Persistent fever	Subjective or documented fever (temperature ≥38°C)	Fever ≥ 3 d
Clinical	All of the following: Single or multiorgan dysfunction (shock, cardiac, respiratory, renal, gastrointestinal, or neurological disorder) Additional features, which might include children fulfilling full or incomplete criteria for Kawasaki disease	All of the following: Clinical severity requiring hospitalization or resulting in death New onset manifestations in at least 2 of the following categories: 1. Cardiac involvement: Left ventricular ejection fraction <55%, or Coronary artery dilatation, aneurysm, or ectasia, or Troponin elevated above laboratory normal range, or indicated as elevated in a clinical note 2. Mucocutaneous involvement: Rash or Inflammation of the oral mucosa (eg, mucosal erythema or swelling, drying or fissuring of the lips, strawberry tongue), or Conjunctivitis or conjunctival injection (redness of the eyes), or Extremity findings (eg, erythema [redness] or edema [swelling] of the hands or feet) 3. Shock 4. Gastrointestinal involvement: Abdominal pain, or Vomiting, or Diarrhea 5. Hematologic involvement indicated by: Platelet count <150 000 cells/μL, or Absolute lymphocyte count (ALC) < 1000 cells/μL	At least 2 of the following: Rash or bilateral nonpurulent conjunctivitis or mucocutaneous inflammation signs (oral, hands or feet). Hypotension or shock. Features of myocardial dysfunction, pericarditis, valvulitis, or coronary abnormalities (including ECHO findings or elevated Troponin/NT-pro-BNP), Evidence of coagulopathy (by PT, PTT, elevated D-Dimers). Acute gastrointestinal problems (diarrhea, vomiting, or abdominal pain).
Inflammation	Evidence of inflammation (neutrophilia, increased C-reactive protein, and lymphopenia)	Evidence of systemic inflammation (indicated by C-reactive protein of ≥3.0 mg/dL [30 mg/L])	Elevated markers of inflammation including ESR, C-reactive protein, or procalcitonin
Evidence of SARS-CoV-2 infection	Positive or negative SARS-CoV-2 PCR test	At least one of the following: Detection of SARS-CoV-2 ribonucleic acid (RNA) in a clinical specimen^d^ up to 60 d prior to or during hospitalization, or in a postmortem specimen using a diagnostic molecular amplification test (eg, polymerase chain reaction [PCR]) Detection of SARS-CoV-2 specific antigen in a clinical specimen^d^ up to 60 d prior to or during hospitalization, or in a postmortem specimen Detection of SARS-CoV-2 specific antibodies^e^ in serum, plasma, or whole blood associated with current illness resulting in or during hospitalization	At least one of the following : Evidence of COVID-19 (positive PCR, or antigen test or serology positive) Likely contact with COVID-19 patients
Exclusion	Exclusion of any other microbial cause, including bacterial sepsis, staphylococcal or streptococcal shock syndromes, and infections associated with myocarditis such as enterovirus.	Absence of a more likely alternative diagnosis. If documented by the clinical treatment team, a final diagnosis of Kawasaki Disease should be considered an alternative diagnosis.	No other obvious microbial cause of inflammation, including bacterial sepsis, staphylococcal, or streptococcal shock syndromes.

^a^Centers for Disease Control and Prevention (CDC) website: https://ndc.services.cdc.gov/case-definitions/multisystem-inflammatory-syndrome-in-children-mis-c-2023/.

^b^World Health Organization (WHO) website: https://www.who.int/news-room/commentaries/detail/multisystem-inflammatory-syndrome-in-children-and-adolescents-with-COVID-19.

^c^Royal College of Pediatrics and Child Health (RCPCH): Harwood et al [72].

^d^Positive molecular or antigen results from self-administered testing using over-the-counter test kits meet laboratory criteria.

^e^Includes a positive serology test regardless of COVID-19 vaccination status. Detection of antinucleocapsid antibody is indicative of SARS-CoV-2 infection, while antispike protein antibody may be induced either by COVID-19 vaccination or by SARS-CoV-2 infection.

To address the diagnostic challenge of distinguishing MIS-C from sepsis, Hernández-García et al developed a differentiation score, MISSEP, based on 5 clinical criteria [77]. These include fever lasting more than 48 hours, thrombocytopenia, abdominal pain, conjunctival erythema, and a vasoactive inotropic score >10 [77]. A score above 25 differentiated MIS-C from sepsis with 89% sensitivity and 95% specificity in the retrospective cohort of Hernández-García et al [77]. Additionally, a study by Avcu et al revealed that infectious causes, predominantly of bacterial origin (71%), are the most frequently [78]. Among these, *Salmonella* spp. and *Campylobacter* spp. Gastroenteritis are notable mimics due to shared symptoms of fever, abdominal pain, and elevated inflammatory markers [79]. However, they lacked true cardiac dysfunction and elevated cardiac biomarkers [79]. Other bacterial infections that may mimic MIS-C include tick-borne illnesses such as Rocky Mountain spotted fever and murine typhus, caused by Rickettsia rickettsii [11, 80–82]. Viral infections such as adenovirus, Epstein–Barr virus, and simple upper respiratory tract infections also present with similar features, although less frequently [78, 83]. Rare, serious disorders, including Stevens–Johnson Syndrome and toxic epidermal necrolysis can also resemble MIS-C [84].

## DISCUSSION

The management of MIS-C involves a multidisciplinary approach aimed at stabilizing the acute inflammatory response and preventing long-term complications. Effective management of MIS-C is critical to preventing severe complications such as cardiogenic shock, coronary artery aneurysms, and multiorgan failure, which can result in long-term morbidity or death if not promptly addressed. Thus, timely management of MIS-C is essential to improving clinical outcomes and helping pave the way for a full recovery.

### Acute-Phase Management

**Figure 1.**
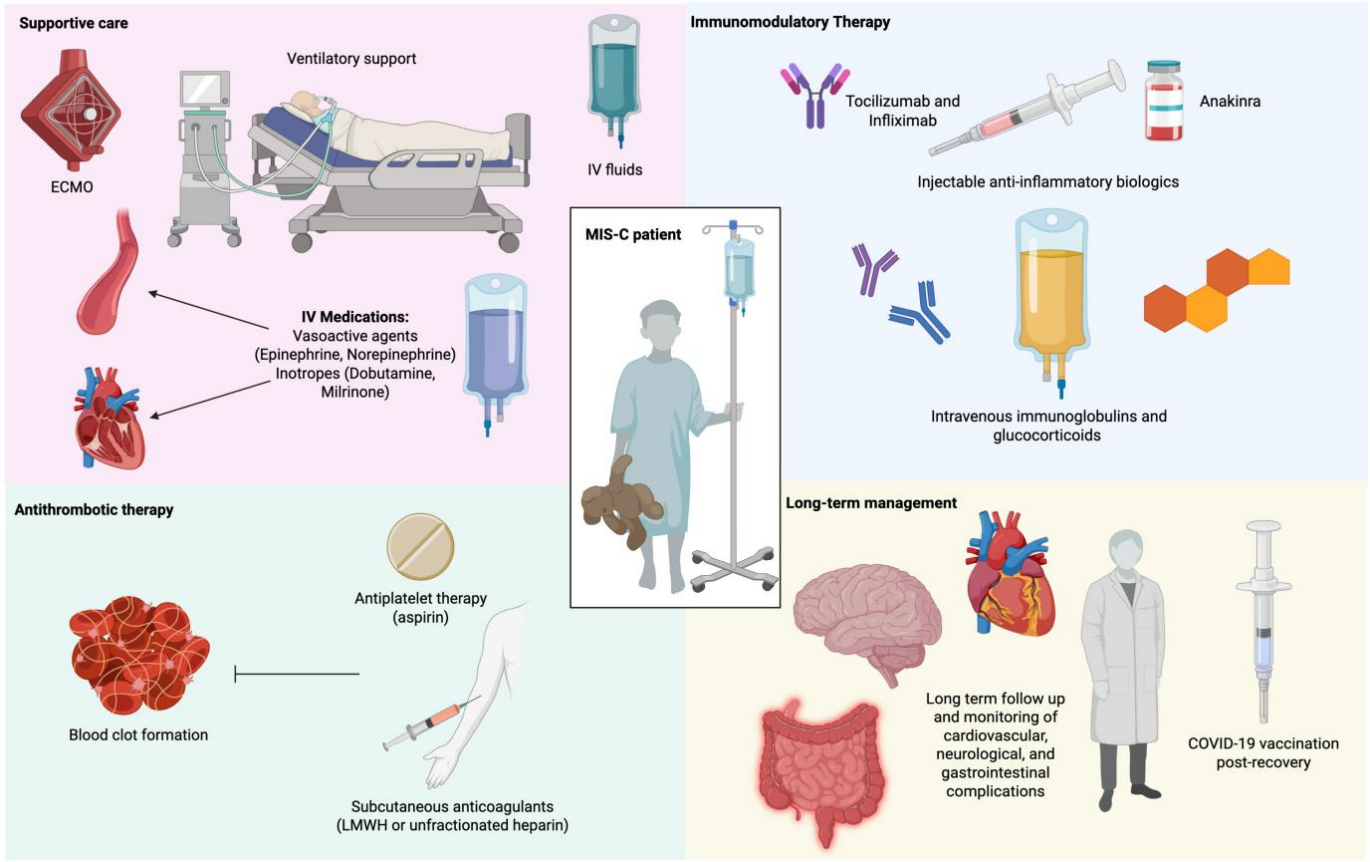
Overview of the multidisciplinary management of multisystem inflammatory syndrome in children (MIS-C). Supportive care (ventilatory support, vasoactive agents, and ECMO) is combined with immunomodulatory therapy, primarily intravenous immunoglobulins and corticosteroids, with biologic agents such as anakinra, tocilizumab, or infliximab reserved for refractory cases. Antithrombotic management (aspirin and low-molecular-weight heparin) addresses the hypercoagulable state, while long-term follow-up targets cardiovascular, neurologic, and gastrointestinal sequelae, alongside postrecovery COVID-19 vaccination. (Figure created on https://BioRender.com).

Initial treatment focuses on supportive care, immunomodulatory therapy, and anticoagulation to mitigate thromboembolic risks. On the other hand, long-term management prioritizes cardiac monitoring and the possibility of COVID-19 vaccination (Figure 1).

#### Supportive Care

Supportive care is a cornerstone in MIS-C management to ensure proper patient stabilization and prevent unwanted inflammation and end-organ damage [85–87]. For MIS-C patients, this includes fluid resuscitation, hemodynamic and ventilation support, which are key to producing favorable outcomes [88]. Fluid resuscitation was found to be effective in children presenting with hypovolemia or distributive shock, with small isotonic boluses best given periodically [64, 89, 90]. Nevertheless, cautious fluid administration is necessary to prevent the development of pulmonary edema, especially in MIS-C patients presenting with myocardial dysfunction [89, 90]. Therefore, these children were often admitted to the PICU (pediatric intensive care unit) when presenting with shock and remained there for close monitoring [91–93].

Vasoactive agents may also be beneficial and aid in the treatment of children presenting with shock. Regarding which vasoactive agents to administer, certain guidelines suggest the use of epinephrine and norepinephrine as opposed to dopamine, with studies showing that the latter was associated with increased mortality and end-organ dysfunction in the pediatric population [94–96]. Moreover, epinephrine is preferred over norepinephrine in MIS-C patients with superimposed myocardial dysfunction, due to its β1-adrenergic effects which enhance cardiac contractility and output [97]. Conversely, norepinephrine primarily increases systemic vascular resistance and afterload, potentially worsening myocardial workload [97]. In children with deteriorating cardiac function and worsening hypotension, an escalation to ionotropic support, using dobutamine or milrinone, was needed [64, 92, 98].

The last aspect of supportive care explored is the need for ventilatory support. Ventilatory support in MIS-C is initiated primarily due to myocardial dysfunction, pulmonary edema, acute respiratory distress syndrome, and shock, which impair oxygenation and increase respiratory distress [34]. Noninvasive support such as high-flow nasal cannula and continuous positive airway pressure/bi-level positive airway pressure, is used for mild-to-moderate cases, while mechanical ventilation is initiated for severe hypoxemia, worsening shock, or respiratory failure, and ECMO is considered in refractory cases [34, 64]. For instance, one meta-analysis showed that a third of patients needed intubation and mechanical ventilation with a majority of these patients having left myocardial dysfunction [99]. In severe cases, ECMO has also been shown to be beneficial in patients presenting with rapid cardiac decompensation, especially with concomitant immunomodulatory and antithrombotic therapy, which will be discussed next [100–102].

#### Immunomodulatory Therapy

Immunomodulatory therapy is crucial in the management of MIS-C, as it helps reduce disease severity by suppressing excessive inflammation, thus improving clinical outcomes. Treatments such as IVIG, corticosteroids, and biologic agents aim to control hyperinflammation and subsequently prevent organ damage and are thus considered to be first-line treatments by most guidelines [19].

According to the ACR, IVIG is now considered a first-line pharmacologic treatment, especially in children with multisystem involvement, systemic inflammation, cardiac dysfunction, or shock requiring inotropic support [19]. The recommended dose is the normally administered dose of IVIG (2 g/kg), often combined with glucocorticoids in severe cases, with careful monitoring of cardiac function and fluid status to prevent cardiovascular complications [19, 103]. Therefore, glucocorticoids are also often considered first-line pharmacologic treatments, with the 2 modalities often going hand in hand to achieve the best results [19, 103]. As reported by several systematic reviews, such a combination resulted in the recovery of critically ill patients [101, 103–105]. The most widely used glucocorticoid in the management of MIS-C is methylprednisone, achieving highly desirable results particularly with earlier initiation [19, 106]. The use of glucocorticoid as a monotherapy may be just as effective as combination with IVIG at a lower cost, especially for milder cases [107]. It is also important for the steroid treatment to be tapered off to prevent a relapse in the inflammatory markers [108].

Biologic therapies are essential in the acute management of MIS-C, as they selectively inhibit key inflammatory pathways—such as IL-1, IL-6, and TNF-α signaling—to modulate the hyperimmune response and prevent multisystem complications [70, 109, 110]. Biologics are notably vital in patients refractory to IVIG and glucocorticoid therapy [16, 19, 64]. The most widely used biologic is anakinra, an IL-1 receptor antagonist, and its use was beneficial even in children without remarkably elevated IL-1 levels, especially if they had myocardial dysfunction [64, 110–114]. Several studies have shown the efficacy of anakinra in treating refractory cases, as anakinra blocks IL-1 signaling, rapidly reduces systemic inflammation and fever, mitigates cardiac dysfunction, and lowers the risk of disease progression [110–114]. Tocilizumab, an IL-6 inhibitor, is another biologic that showed promise in the treatment of refractory cases of MIS-C, decreasing the hospital stay compared with an increase with anakinra use [64, 115, 116]. In addition to the usual desired effects of biologics, tocilizumab was capable of alleviating the cytokine storm associated with COVID-19 and its subsequent symptoms [117–119]. Last of all, infliximab, a TNF-α inhibitor, has been used in refractory MIS-C as well, reducing inflammation and aiding in the restoration of cardiac function [64, 120–123]. Its anti-inflammatory effects may also help reduce the risk of coronary artery aneurysms by suppressing vascular inflammation and preventing endothelial damage [108, 124].

The selection of biologic therapy in MIS-C is guided by the predominant inflammatory pathway, disease severity, and response to initial treatment. Anakinra is generally preferred as the “first-line biologic” in patients refractory to IVIG and corticosteroids, particularly when myocardial dysfunction or macrophage activation features are present [125, 126]. Tocilizumab or infliximab may be considered when IL-6– or TNF-α–driven inflammation predominates or if there is inadequate response to IL-1 blockade [125, 127].

#### Antithrombotic Therapy

Antithrombotic therapy is the last pillar of treatment that has been used to prevent serious thrombotic complications in MIS-C patients [128]. Two types of antithrombotic medications were used: anticoagulant and antiplatelet drugs. Anticoagulants like enoxaparin or unfractionated heparin are used prophylactically to prevent and treat thrombosis due to the hypercoagulable state seen in MIS-C patients [129–134]. They were particularly relevant in patients with elevated D-dimer, cardiac dysfunction, or confirmed clot formation [131–134]. Importantly, major bleeds as a result of treatment were a highly unlikely outcome [130, 131]. With regards to antiplatelet therapy, low-dose aspirin was most commonly used to reduce the risk of coronary artery thrombosis, especially in cases with Kawasaki-like features [99, 135–137]. Thus, the combination of anticoagulants and antiplatelet therapies is essential to avoid serious cardiovascular manifestations associated with thrombotic events.

### Long-Term Management

Children recovering from MIS-C require careful long-term monitoring to prevent and manage potential complications affecting multiple organ systems. Additionally, postrecovery vaccination, particularly regarding the safety and timing of the COVID-19 vaccine, is a key consideration in protecting these children from future infections while minimizing potential risks.

#### Monitoring for Other Sequelae

Long-term management of MIS-C focuses on cardiac, neurological, and gastrointestinal follow-up to detect and monitor their sequelae. A multidisciplinary approach involving cardiology, neurology, gastroenterology, and rehabilitation specialists is optimal. Cardiac monitoring is crucial, especially for serious entities such as CAA and myocardial dysfunction [26, 138, 139]. Ideally, echocardiograms should be performed at 1–2 weeks, 4–6 weeks, 3 months, and 12 months [138, 140]. Moreover, patients with small aneurysms may need to continue low-dose aspirin for at least 6 weeks, whereas larger aneurysms may require anticoagulation therapy such as enoxaparin or warfarin [138]. With regards to physical activity, exercise should be restricted for 3–6 months, with clearance via cardiac MRI or stress testing before resumption [140].

Neurological and gastrointestinal problems are usually less severe and less common than the cardiac complications resulting from MIS-C. Nevertheless, close monitoring and observation is still required. Long-term neurological and psychological manifestations may significantly impact quality of life [22, 141]. Therefore, severe headaches, cognitive fog, mood disturbances, and depression symptoms need to be detected and subsequently managed by performing regular neurology and psychological assessment [19, 22, 142, 143]. Severe cases may require brain EEG and MRI, and cognitive rehabilitation may help children with neurocognitive deficits [144, 145].

Gastrointestinal symptoms may also persist for a prolonged period of time after acute resolution of MIS-C [141, 146]. Persistent inflammation of the gastrointestinal system was observed in some children weeks after an acute MIS-C flare up [146, 147]. Furthermore, metabolic disturbances may persist, with observations of insulin resistance and altered glycometabolic markers up to a year post-MIS-C [141, 148]. Thus, continued follow-up, including imaging, liver function tests, inflammatory marker screening, and possible gastroenterology consultation is recommended [149]. Metabolic monitoring, such as glucose tolerance testing and lipid panels, is also recommended, as some children develop insulin resistance [148]. Taken together, a multidisciplinary approach involving at least cardiology, neurology, and gastrointestinal follow-ups ensures early intervention in case of complications and better long-term outcomes.

#### Vaccination Postrecovery

COVID-19 vaccination after recovering from MIS-C is an important aspect of long-term care, helping prevent complications and, more importantly, reduce the risk of another SARS-CoV-2 infection that could trigger a recurrence of MIS-C. As detailed by the CDC, children must have fully recovered from MIS-C and wait at least 90 days from the diagnosis or first appearance of symptoms to be eligible for the vaccine [150]. The vaccine is still recommended in these children despite the risk of myocarditis, as in these cases, the benefits outweigh the risks [150]. In children with a history of MIS-C who received the vaccine, the vast majority of adverse events were minor, including arm soreness and fatigue, similar to what is observed in the general population [151–153]. Following vaccination, these children had a significantly boosted antibody response to the virus spike protein that was maintained for up to 3 months [154]. A brief mention of COVID-19 vaccination in healthy children and its association with MIS-C development is appropriate. Some studies have shown that MIS-C may develop postvaccination in some children, warranting the question of whether a child should receive the vaccine out of concern for developing MIS-C [151, 155]. Nonetheless, this scenario is exceedingly rare, and the risk of developing MIS-C following a SARS-CoV-2 infection is significantly higher than after receiving the vaccine [151, 156]. Therefore, the vaccine remains a crucial element of long-term management of MIS-C and is even indicated in MIS-C prevention in previously healthy children.

### Challenges in Management

One pivotal aspect of managing MIS-C that precedes providing proper treatment is the timely detection of MIS-C. As seen throughout, MIS-C has a wide range of clinical manifestations, and the lack of a unified definition for the disease further complicates diagnosis [157]. MIS-C is also very commonly misdiagnosed, possibly masking malignancies, infections, or other inflammatory diseases [78]. Van der Steen et al describe 3 cases that were initially diagnosed with MIS-C, as all met the criteria set by the RCPCH, with some also meeting the WHO and CDC criteria [157]. However, it was later found that although the criteria were fulfilled, they were not true MIS-C cases, with sepsis, IBD (inflammatory bowel diseases), and perforated appendicitis as the etiologies of their presentation [157]. Misdiagnosing MIS-C may also lead to unnecessarily inflicting potentially harmful side effects and financial loss due to expensive management modalities [157]. Misdiagnosis of MIS-C can be detrimental because it delays the initiation of appropriate immunomodulatory therapy and supportive care, allowing persistent hyperinflammation and potential multiorgan injury due to unchecked immune dysregulation [24]. For this reason, any suspected MIS-C case requires a multidisciplinary approach [88].

## CONCLUSION

MIS-C is a rare but potentially life-threatening complication of SARS-CoV-2 infection in the pediatric population. While its clinical presentation can mimic other inflammatory and infectious conditions, prompt recognition and differentiation are critical to initiating appropriate treatment. Although significant advances have been made in understanding the pathophysiology and optimal management strategies including immunomodulatory, antithrombotic, and supportive therapies, a unified diagnostic framework and treatment algorithm remain lacking. As our understanding of MIS-C continues to evolve, further research is needed to refine diagnostic tools, standardize treatment protocols, and optimize long-term outcomes for affected children.
